# The roles of ribosomal proteins in nasopharyngeal cancer: culprits, sentinels or both

**DOI:** 10.1186/s40364-021-00311-x

**Published:** 2021-06-30

**Authors:** Edmund Ui-Hang Sim, Choon-Weng Lee, Kumaran Narayanan

**Affiliations:** 1 Faculty of Resource Science and Technology, Universiti Malaysia Sarawak, 94300 Kota Samarahan, Sarawak Malaysia; 2 Institute of Biological Sciences, University of Malaya, 50603 Kuala Lumpur, Malaysia; 3 School of Science, Monash University, 46150 Bandar Sunway, Selangor Malaysia; 4 Department of Genetics and Genomics Sciences, Mount Sinai School of Medicine, New York, NY 10029 USA

**Keywords:** Ribosomal proteins, Nasopharyngeal carcinoma, Carcinogenesis, Cancer genetics, Medical genetics

## Abstract

Ribosomal protein genes encode products that are essential for cellular protein biosynthesis and are major components of ribosomes. Canonically, they are involved in the complex system of ribosome biogenesis pivotal to the catalysis of protein translation. Amid this tightly organised process, some ribosomal proteins have unique spatial and temporal physiological activity giving rise to their extra-ribosomal functions. Many of these extra-ribosomal roles pertain to cellular growth and differentiation, thus implicating the involvement of some ribosomal proteins in organogenesis. Consequently, dysregulated functions of these ribosomal proteins could be linked to oncogenesis or neoplastic transformation of human cells. Their suspected roles in carcinogenesis have been reported but not specifically explained for malignancy of the nasopharynx. This is despite the fact that literature since one and half decade ago have documented the association of ribosomal proteins to nasopharyngeal cancer. In this review, we explain the association and contribution of dysregulated expression among a subset of ribosomal proteins to nasopharyngeal oncogenesis. The relationship of these ribosomal proteins with the cancer are explained. We provide information to indicate that the dysfunctional extra-ribosomal activities of specific ribosomal proteins are tightly involved with the molecular pathogenesis of nasopharyngeal cancer albeit mechanisms yet to be precisely defined. The complete knowledge of this will impact future applications in the effective management of nasopharyngeal cancer.

## Background

Eukaryotic ribosomal proteins (RPs) comprises 79 different known types that are broadly divided into two groups, the small (40S) and large (60S) ribosomal subunits. Since 2014, a revised naming system for RPs was published [1] and this is used in this review. In this improved alphanumeric nomenclature system, the prefixes eS, and uS connote eukaryotic and universal ribosomal proteins of the small subunit respectively. The prefixes eL, and uL connote eukaryotic and universal ribosomal proteins of the large subunit respectively.

Interestingly, albeit within an integrated system of transcriptional and translational regulation, some extent of uniqueness occurs among RPs in the defined physiological regulation of specific genes [2]. This gave rise to the cogent suspicion that RPs have physiological significance extraneous to ribosome biogenesis and protein biosynthesis. Indeed, as early as the mid-90s, evidence emerged to explain the extra-ribosomal functions of RPs that include DNA replication, transcription, DNA repair, DNA splicing and modification, and apoptosis [3]. Since then, there has been a steady increase in reports or findings documenting these extraneous functions of RPs [4–6] as listed in Table 1. The tight relationship of ribosomal proteins with cell development and differentiation through their extra-ribosomal functions also means that any altercation of their structures and/or expression can result in mal-development and malignancy. The physiological connection between RPs and cancers has also been extensively reviewed and explained [5, 6], including their interaction with the p53-MDM2 complex in events of carcinogenesis [7]. The focus of this review is confined to the relationships of RPs with nasopharyngeal carcinoma (NPC). This cancer begins as a malignant tumour at the epithelial lining of the nasopharynx, more precisely at the Fossa of Rosenmuller – a depression next to and above the opening of the Eustachian tube [8, 9]. A comprehensive review on NPC-associated RPs (NRPs) and their significance in the NPC oncogenesis is timely to facilitate further endeavours on exploring NRPs as targets for diagnosis and prognostic biomarkers, and targeted drug therapy.

**Table 1:** Known possible extra-ribosomal functions of eukaryotic ribosomal proteins as derived from Warner and McIntosh, [4]; de las Heras-Rubio et al., [5]; Xu et al., [6]

Ribosomal proteins	Extra-ribosomal functions
eL30, uS14, uL12, uS13	Inhibits its own pre-mRNA splicing
uL2, eS28	Shortens its own mRNA T_1/2_
uL18, uL5, uL14, uL24, eS7	Sequesters M/HDM2 from ubiquitinizing E3
uL5	Sequesters c-Myc from transactivating its targets
uL24	Promotes p53 translation
uL14	Negatively regulate Miz1 by sequestering nucleophosmin
RACK1	Cell signalling via acting as a receptor for protein kinase C
uL13	Inhibits mRNA translation (GAIT complex) subset of inflammation-related proteins
uS3	Act as a DNA endonuclease (apurinic/apyrimidinic endonuclease III) for DNA repair; binds NFkB; and serves as a signal mediator between neuronal apoptosis and DNA repair
uL16	Binds c-jun
uS10, eL6	Influences Pol III transcription
eL22	Binds Histone H1 (affects transcription), and forms a RNP with Epstein–Barr-encoded small RNA (EBER-1) in B lymphocytes
eS26	Susceptibility factor to diabetes
uS10	Participates in anti-termination by RNA polymerase III
uL3	Induction of G1/S arrest or apoptosis by modulating p21
uL10	DNA repair: apurinic/apyrimidinic endonuclease III; promotes viral infection; and functions in viral translation
uS11	Negatively controls splicing of its own pre-mRNA
uS15	Negatively controls splicing of its own pre-mRNA
uL30	Inhibits the translation of specific mRNAs, including its own
eL19	Regulation of the Slit-Robo signalling pathway for axon guidance and angiogenesis
eS1	Modulation of erythropoiesis, and binds to the Epstein Barr virus encoded protein EBNA5
P2	Iron-binding protein responsible for distributing iron intracellularly

As such, this review covers what has been known thus far from the link between RPs and NPC, and what has been proposed regarding the molecular pathogenesis mediated by NPC-associated RPs (NRPs) in NPC situation. Literature reviewed here encompasses findings on cancer-associated RPs, current knowledge on NPC and NRPs, and information on the plausible biological roles of NRPs in the context of NPC carcinogenesis. Issues under discussion include the complex relationship between NRPs and NPC that highlights the complexities on the former’s roles and mechanisms in the neoplasm of the latter; and the applicability of NRPs as biomarkers for NPC.

### Ribosomal proteins and cancers

Early evidence of the association between RPs and cancers came from the observations of haploinsufficiency of eS4 in Turner Syndrome [10] and *eS19* mutation in the Diamond-Blackfan Anaemia (DBA) condition [11]. Besides *eS19*, mutations and deregulation of several other RP genes have been reported to be associated with cancer in DBA individuals [12]. In colorectal cancer, numerous RP genes were reportedly dysregulated [13, 14] suggesting their roles in the regulation of cell proliferation, apoptosis, tumor suppressors, and malignant transformation/progression [15]. Besides colorectal malignancy, association of RPs to cancers includes uL14 in lung adenocarcinoma [16]; eL22 in T-cell acute lymphoblastic leukemia [17]; eL8, eL37, eS19, eS21, eS24, and eS27 in prostate cancer [18–20]; uS8 in breast cancer [21], eS27 in gastric carcinomas [22]; *eL5* and *eL14* in ovarian cancer [23]; and uS8 and RACK1 in liver cancer [24, 25]. Table 2 provides an overview of RP-associated cancer-related processes based on information from Xu et al. [6]

**Table 2:** Plausible roles of RPs in tumorigenesis

Ribosomal proteins	Cancer-related processes
uS3, eS1, eS6, eS7, uS11, eS25, eS27, uS14, uL3, eL6, uL30, uL2, uL14, uL24	Apoptosis
uS3, uS7, eS6, eS7, uS11, uS19, eS19, eS10, eS25, eS26, eS27, eS31, uL3, uL18, eL6, uL30, uL5, eL13, uL14, uL24, eL31, eL34, eL37, eL41	Cell cycle
eS6, uS4, uS15, uS11, uS8, eS24, eS27, eL6, uL2, uL5, eL15, uL22, uL24, eL29, eL31, eL34, eL42	Cell proliferation
P1, eS1, uS11, uL18, eL22, eL41	Neoplastic transformation
uS3, eS6, eS7, uS8, eS24, eS27, eL15	Cell migration and invasion

### Nasopharyngeal carcinoma (NPC)

NPC patients present with a wide range of symptoms and are usually confirmed upon histopathological examination of tissue biopsies [26]. The World Health Organisation (WHO) classification of NPC constitutes three major types, that is the Type I, II, and III [27] with Type II being the most common [28]. NPC has moderate to high prevalence in Southern China, Southeast Asia, Arctic and North Africa [29–33] and particularly among the Cantonese in China [32, 33]. Early indication of genetic susceptibility to NPC came from the Human Leucocyte Antigen (HLA) factor [34]. This is followed by reports of allelic loss in chromosome 3p, 11q, and the inactivation of *RASSF1A* [35–37]. Besides this, a correlation between NPC pathogenesis and Epstein-Barr Virus (EBV) infection has been established [38] with higher EBV positivity in Type II compared to Type I NPC [39]. Environmental factors such as the over-consumption of salt-preserved food [40–42], cigarette smoking [40, 41, 43], and cumulative exposure to formaldehyde [44] have been reportedly linked to the increased risk of NPC. Almost all NPC scenarios begin with EBV infection, but the concerted roles of genetic factors, viral infection, and environmental triggers are necessary for the manifestation of the disease.

### Diagnosis and treatment of NPC

NPC is one of the most misdiagnosed cancers whereby a majority of reported cases are from advanced stages with poor prognosis. Only 9% of cases are detected at Stage I, while 83 and 39% at Stages II/III, and IV respectively [45]. Conventional diagnosis is by nasopharyngeal endoscopy, lymph node histopathology, and immunoassay of EBV-derived antigens [46]. Biomarkers such as Galectin-1 [47], SRY-related HMG-box 4 (SOX4) [48], CXC chemokine receptor type 7 (CXCR7) [49], hypoxia up-regulated 1 (HYOU1) [50], Kelch Domain Containing 4 (KLHDC4) [51], Aldo-keto-reductase 1B10 (AKR1B10) [52], prohibitin-1 (PHB1) [53], and Cyclooxygnenase 2 (Cox-2) [54] have also been identified. A combined approach of using the C-C motif chemokine ligand 27 (CCL27) biomarker and EBV-associated antigens can increase detection sensitivity [55]. Treatment of NPC depends on the location and invasiveness of the tumor, as well as the patient’s overall health status. Early non-metastatic stages (in situ tumors) is usually treated using the intensity-modulated radiotherapy (IMRT) [56]. Advanced stages are often managed using radiotherapy and chemotherapy (docetaxel, cisplatin or 5-fluorouracil) [57]. Recently, the molecule-based targeted therapy using Cetuximab (a chimeric monoclonal antibody that targets and inhibits the epidermal growth factor receptor, EGFR) concurrently with induction cisplatin-based chemoradiotherapy has significantly increased the overall survival rate of patients [58].

### Ribosomal proteins and nasopharyngeal carcinoma

Initial findings of NPC-associated RP (NRP) were revealed in the elevated expression of metallopanstimulin 1 (MPS-1) in head and neck malignancies [59] – an RP encoded by the *eS27* gene [60]. The transcript levels of *eS27* and *eS26* have also been found to be down-regulated in NPC tissues [61]. Hence, besides establishing eS27 as the first NRP, an additional NRP (eS26) was identified. This baited the question on the full repertoire of NRPs. Indeed, a study by Fang and co-workers [62] revealed the transcripts of *uS7* and *uS19* to be up-regulated in NPC tissues. It seems that the aberrant expressions of selected RP genes are connected with NPC tumorigenesis. The analysis of 18 RP genes of the large ribosomal subunit component by comparing their expression pattern between NPC cell lines (derived from keratinising-differentiated and non-keratinising-poorly differentiated squamous cell carcinoma tumours of the nasopharynx) and normal control uncovered three RP genes (*eL27*, *eL43*, and *eL41*) to be significantly down-regulated in the NPC cell lines [63]. However, a subsequent study revealed these three RP genes to be markedly over-expressed, in terms of transcript and protein levels, in NPC cell lines compared to normal control [64]. These conflicting results raised more confusion over the nature of the expression pattern of NRP genes between different studies. Nevertheless, in a later study involving more cell lines, four more RP genes (*uS8*, *uS4*, *eS31*, and *uL14*) that are differentially expressed between cancer and normal cell lines were discovered [65]. These were down-regulated in NPC cell lines rather than up-regulated. Finally, in a most recent study, the down-regulation of *eL14* and up-regulation of *uS19* in NPC cell lines relative to normal control were reported [66]. This brings the repertoire of NRP genes to 12, comprising 5 and 7 members from the large and small ribosomal subunits respectively. Four and five RP genes are categorically up-regulated and down-regulated respectively, while four other RP genes are arguably inconsistent between studies. Table 3 summarises the latest list of NRP genes.

**Table 3:** List of differentially expressed RP genes in the context of NPC tumourigenesis

Ribosomal subunit	RP genes	Expression level	NPC model	References
Large (60S)	*eL14*	Up-regulated (transcript)	Cell lines	[66]
	*uL14*	Down-regulated (transcript)	Cell lines	[65]
	*eL27*^*a*^ *eL41*^*a*^ *eL43*^*a*^	Down-regulated (transcript)	Cell lines	[63]
	*eL27*^*a*^ *eL41*^*a*^ *eL43*^*a*^	Up-regulated (transcript and protein)	Cell lines	[64]
Small (40S)	*uS4*	Down-regulated (transcript)	Cell lines	[65]
	*uS7*	Up-regulated (transcript)	Tissues	[62]
	*uS8*	Down-regulated (transcript)	Cell lines	[65]
	*uS19*	Up-regulated (transcript)	Cell lines and tissues	[62, 66]
	*eS26*	Down-regulated (transcript)	Tissues	[61]
	*eS27*^*a*^	Up-regulated (protein)	Tissues	[59]
	*eS27*^*a*^	Down-regulated (transcript)	Tissues	[61]
	*eS31*	Down-regulated (transcript)	Cell lines	[65]

^a^Ribosomal protein genes that showed inconsistency in expression patterns between studies

Despite the strong association of RPs with NPC carcinogenesis, little is known about their mechanism(s) in the malignancy. A problem here is their inconsistent expression patterns between different studies (Table 3). For example, early studies on *eS27* [59] revealed its up-regulation in NPC tissues relative to normal nasopharyngeal tissues. A subsequent study showed that it was down-regulated instead in NPC tissues [61]. Compounding this was a later study [67] that nullified both *eS27* and *eS26* to be linked to NPC tumorigenesis. Similarly, the narrative of *eL27*, *eL41*, and *eL43* changes when studied at different period despite using the same cancer models [63, 64]. This phenomenon is difficult to elucidate and indicates the complex relationship between RP genes and NPC malignancy.

The disparity in expression patterns among the different NRP genes suggesting their unique behaviours in NPC. Some NRPs are up-regulated while others are down-regulated (Table 3). There is no regular or predictable pattern. Is this irregularity due to their specialised activities during organogenesis? A possible answer to this is the fact that an intricate level of specialisation exists among RPs in the precise regulation of specific genes during cellular processes [2]. Since the activities of each NRP differ from one another, their dysregulation (albeit varied in nature) could concertedly contribute to carcinogenesis.

Another concern in the expression pattern of NRPs is that many of the findings are based on transcript (mRNA) levels. There is a possibility that post-translational control involving the rapid degradation of surplus NRPs may balance the effects of differential transcript levels. However, a parallel pattern between differentially expressed mRNAs and proteins of three RP genes has been observed in the NPC cell lines [64]. In fact, in an in vivo study to compare mRNA and protein levels in an ovarian cancer model, differentially expressed mRNAs did correlate significantly with their protein products – better than in situation involving non-differentially expressed mRNAs [68]. Therefore, interpreting differentially expressed transcripts of selected RPs as NRPs is still relevant. Nevertheless, further studies to compare the mRNA and protein levels of all NRPs are needed to establish this relationship.

### Putative partners of ribosomal proteins in NPC scenario

Before the discovery of NRPs, a preliminary indication of RP-linked NPC oncogenesis came from the observation of the association between eL22 and EBV. eL22 binds to one of the EBV-encoded RNAs, EBER-1 [69] and EBERs have been known to enhance the proliferative capability of NPC cells [70]. Therefore, a role for EBV in NPC oncogenesis via the agency of NRPs is logical, specifically via the eL22-EBER-1 complex. In Burkitt’s Lymphoma cell lines, elevated proliferation was attributed to the sequestration of eL22 by EBER-1 and its subsequent relocalisation from nucleoli to nucleoplasm [71]. Whether these events are similar in NPC scenario remain to be explored.

**Fig. 1:**
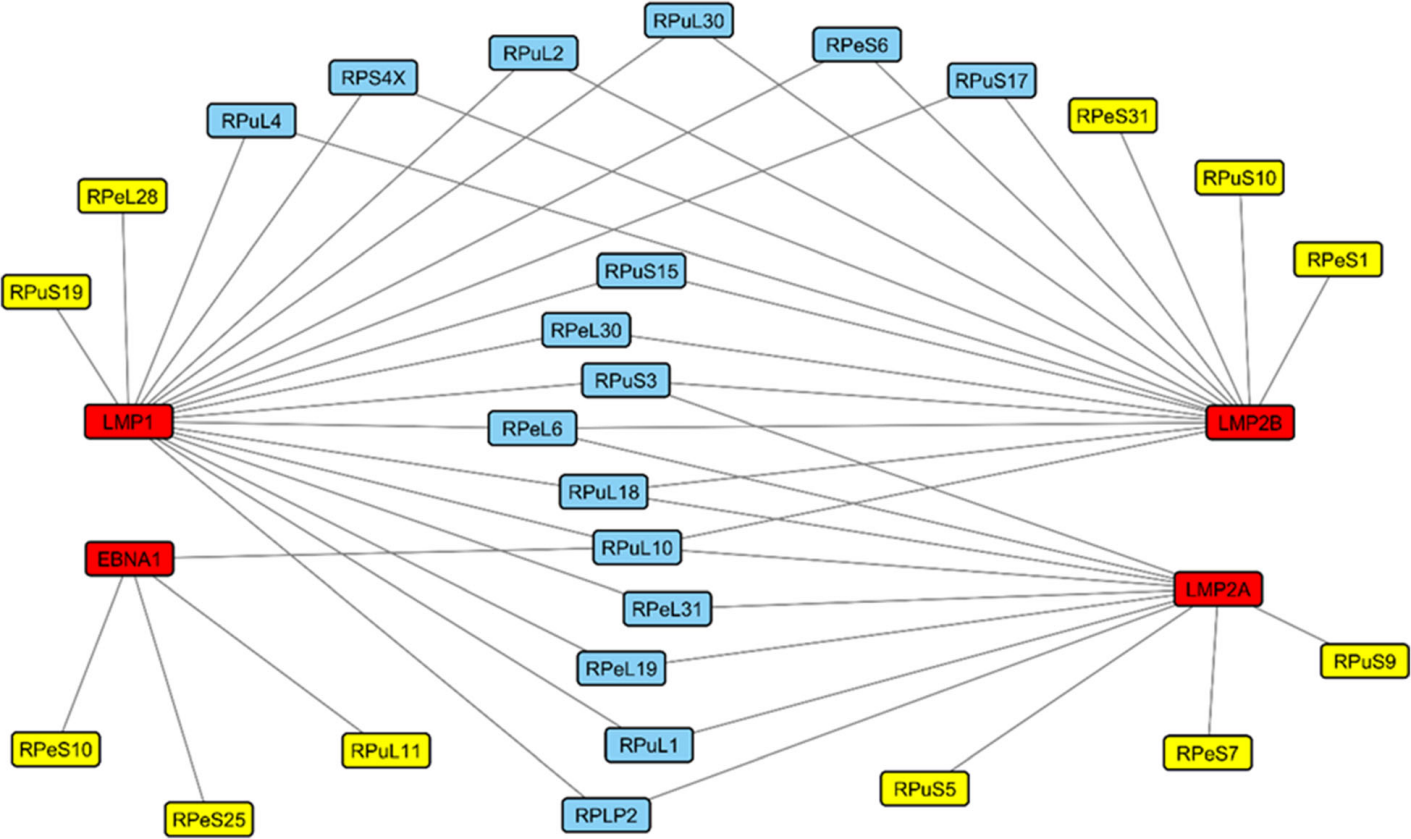
Computationally predicted interactions between EBV and ribosomal proteins (taken from Sim & Talwar, [81])

In the case of protein partners of NRPs, a prospective scenario is the RP-MDM2-p53 pathway. The tumour suppressor, p53 plays a pivotal function in cellular stability in response to nucleolar stress and is negatively regulated by a few factors, one of which is the Mouse Double Minute 2 homolog (MDM2) protein [72]. Incidentally, MDM2 interact directly with several types of RP such as uL4, uL5, uL14, uL18, uL24, eS7, and eS25 [73–75]. Except for uL24, these RPs bind to MDM2 to inhibit its function of ubiquitination and degradation of p53 during events of cellular stress. Conversely, uL24 is a direct translational activator of p53, and is itself negatively regulated by MDM2 [75]. *p53* is the most frequently mutated gene in NPC [76] with specific mutation able to confer its oncogenic potential in NPC cells [77]. It is also linked to poor prognosis and worse survival rate of NPC patients, while MDM2 expression correlates with distant metastasis [78]. The connection between EBV infection and p53 expression in NPC oncogenesis [79, 80] may also include the hypothetical RP-MDM2-p53 pathway. In fact, there is now in silico evidence of plausible logical interactions between four EBV-related proteins with a myriad of RPs (Fig. 1) [81]. More specifically, the functional interactions between the Epstein–Barr nuclear antigen 1 (EBNA1) protein with four RPs individually via the complexes of EBNA1-eS10, EBNA1-eS25, EBNA1-uL10, and EBNA1-uL11 have been predicted. These are pertinent information not because EBNA1 is the only EBV protein found in all EBV-related malignancies [82, 83] but because it is the first time an EBV-encoded protein is suspected to be associated with RPs. Although the biological relevance of these hypothetical interactions to NPC oncogenesis requires experimental proof, the most plausible candidate is the EBNA1-eS25 complex.

**Fig. 2:**
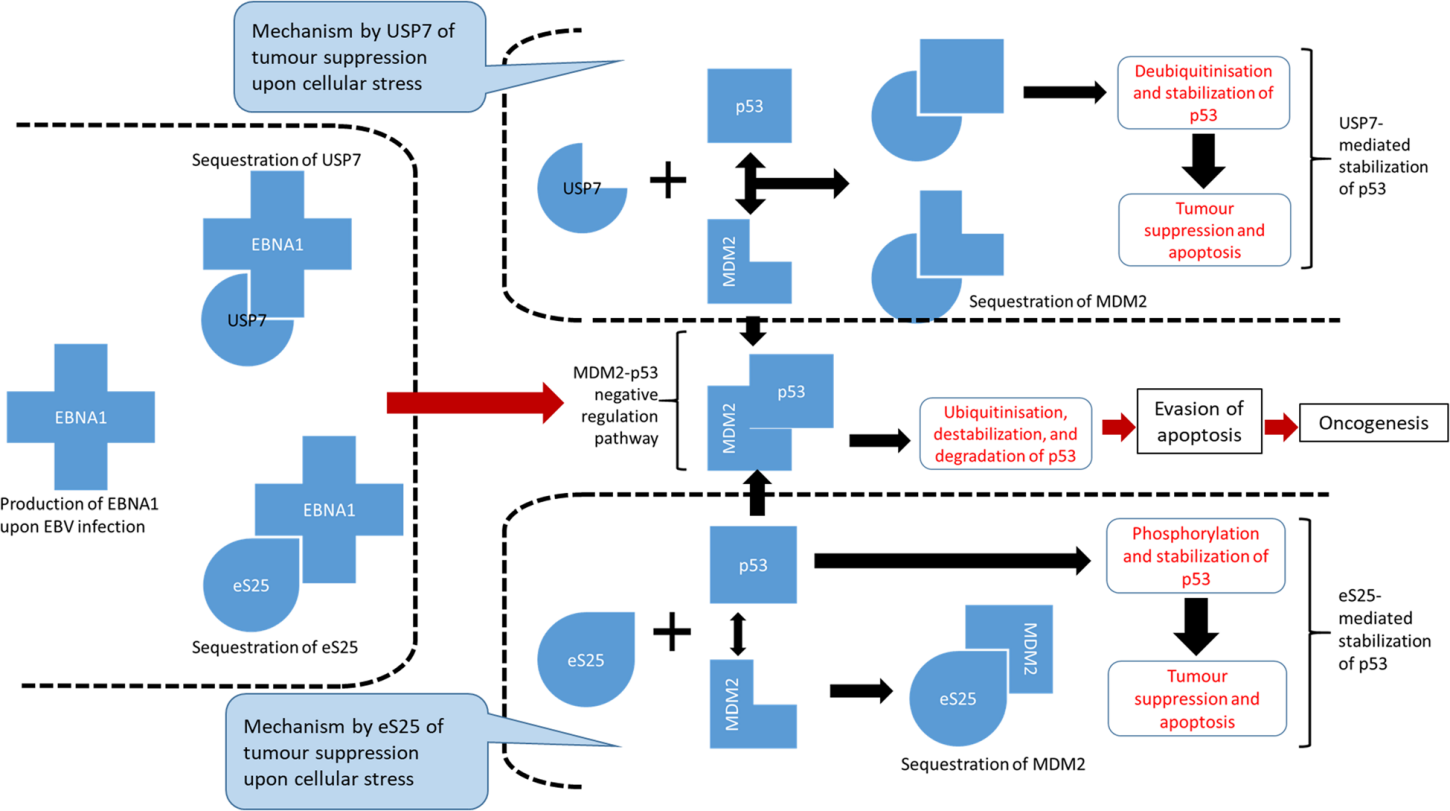
Schematic diagram of the hypothetical elucidation on the roles of EBNA1, eS25, and USP7 in NPC oncogenesis

EBNA1 binds with the cellular ubiquitin-specific protease (USP7/HAUSP) [84, 85], in the same site as that recognised and bound by p53 and MDM2 [86]. In a way, EBNA1 competes with p53/MDM2 in binding with USP7. The interaction between USP7 and p53/MDM2 affects the de-ubiquitination and stabilisation of p53 [87, 88]. When EBNA-1 binds to USP7 the latter is sequestered by the former thereby creating an environment where p53 cannot be stabilised (Fig. 2). eS25 has also been shown to bind to MDM2 and subsequently inhibiting MDM2 from destabilising p53 [74]. The sequestration of MDM2 by eS25 facilitates the activation and stabilisation of p53 (Fig. 2). eS25 and USP7 have the same effect on the MDM2-p53 pathway with the outcome of a stabilised p53. In the EBNA1-USP7 scenario, a direct interaction between EBNA1 and USP7 has been experimentally proven [84]. The interaction between EBNA1 and eS25 is, however, only computationally predicted and requires experimental verification. Moreover, the direct association between EBNA1 and the MDM2-p53 complex is yet to be determined. It seems that the only way for EBNA1 to abrogate tumour suppression by p53 is via intermediary factors. Both USP7 and eS25 fit the description of such intermediary factors. A hypothetical elucidation of their roles in NPC oncogenesis is illustrated in Fig. 2.

Another relevant narrative based on our computational analysis is the predicted interaction between the EBV-encoded latent membrane protein 1, LMP1 and the RP, uS19 (Fig. 1). LMP1 is the principal viral oncoprotein of EBV [89] and is expressed in many human malignancies [90], including NPC [91]. The *uS19* transcript is overexpressed in NPC tissues [62] and cell lines [66]. The speculated interplay between LMP1 and uS19 during NPC oncogenesis can be anecdotally construed from literature other than their overexpression in NPC tissues/cells. LMP1 has been known to affect the normal functioning of p53 via various mechanisms. These include the inhibition of p53-mediated apoptosis through induction of the TNFAIP3/A20 pathway [92], phosphorylation-associated modification of p53 activity through the activation of the MAPK/SAPK pathway [93], overriding tumour suppressor activity of p53 by synergising with Bcl-2 [94], and triggering expression of MDM2 to induce p53 degradation [95]. For uS19, its role in the activation of p53 via direct interaction with MDM2 has been reported [7]. By directly binding to MDM2, the E3 ubiquitin ligase activity of MDM2 is inhibited leading to p53 stabilisation. Combining literature knowledge of LMP1 and uS19 in this respect, we speculate that upon EBV infection of nasopharyngeal epithelial cells, LMP1 influences a series of molecular events that destabilises p53 including removing the regulatory role of uS19 in the MDM2-p53 pathway.

**Fig. 3:**
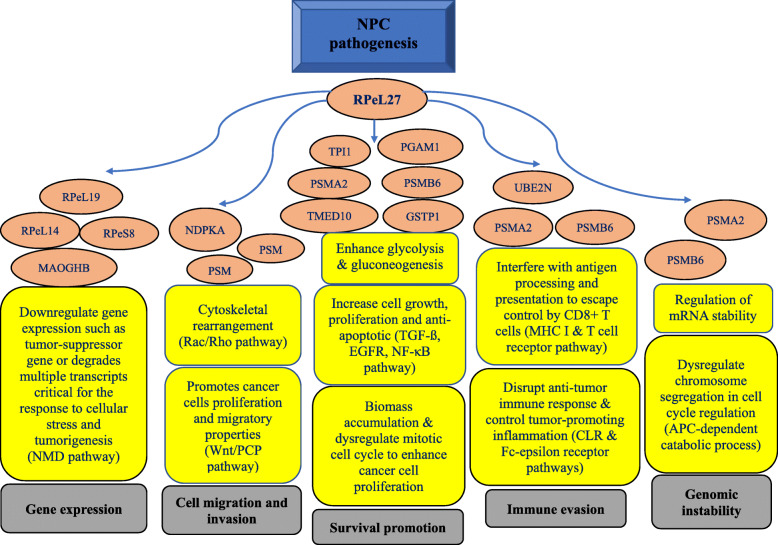
A flowchart of NPC pathogenesis that is associated with the activities of the ribosomal protein, eL27 (taken from Sim & Yew [96])

The latest discovery on the potential pathways mediated by NRP involves the eL27 protein. Initially, the mRNA transcript of this NRP was found to be down-regulated in NPC cell lines [61] but later discovered its transcript and protein to be elevated [64]. Most recently and importantly, from a deeper analysis that included gene knockdown, protein profiling, and bioinformatics, 15 possible interacting partners of eL27 and their plausible roles in the pathogenesis of NPC (Fig. 3) were identified [96].

### Future outlook

The connection between RPs and NPC is an established relationship not only because a sizable list of NRPs is available, but also that several putative RP-mediated pathways relevant to NPC malignancy are evident. This account is crucial in the prudent interpretation of the molecular basis of NPC. Biomedical applications will benefit immensely from this. Studies on chemical and molecular inducers/inhibitors of NRPs can be explored as one of the treatment regimes. Also, an NRP-based platform for molecular diagnosis and prognosis of NPC can be developed. Despite current advances in the understanding of NRPs, knowledge of the complex biochemical networks and molecular events mediated by them during NPC malignancy is still insufficient. It is because the expression behaviours of some NRPs are still elusive. In addition, more studies that look into their protein (rather than just transcript/mRNA) activity levels and functions will be required to firmly establish the nature of their relationship with NPC tumorigenesis. Whether NRPs can be labelled as culprits or sentinels or both in the context of NPC oncogenesis is unclear at the present moment. Deriving a definitive NRP-mediated pathway underlying the pathogenesis of NPC pathogenesis will ultimately require more extensive and in-depth studies.

## Conclusions

Expression, functional, and bioinformatics studies over the years have cumulatively provided a considerable repertoire of NRPs and multiple proposed pathways. These provide essential insights into the molecular narrative of nasopharyngeal cancer that will aid future biomedical innovation in managing this disease. Nevertheless, in tandem with potential translational research, fundamental studies on the NRP-mediated molecular pathogenesis of NPC remain vital.

## Data Availability

Not applicable.

## Abbreviations

AKR1B10: Aldo-keto-reductase 1B10
Bcl-2: B-cell lymphoma 2
CCL27: C-C motif chemokine ligand 27
Cox-2: Cyclooxygnenase 2
CXCR7: CXC chemokine receptor type 7
DBA: Diamond-Blackfan Anaemia
DNA: Deoxyribonucleic acid
EBER: EBV-encoded small RNA
EBNA: Epstein–Barr nuclear antigen
EBV: Epstein-Barr Virus
EGFR: Epidermal growth factor receptor
HAUSP: Herpesvirus-associated ubiquitin-specific protease
HLA: Human Leucocyte Antigen
HMG: High mobility group
HYOU1: Hypoxia up-regulated 1
IMRT: Intensity-modulated radiotherapy
KLHDC4: Kelch Domain Containing 4
LMP: Latent membrane protein
MAPK/SAPK: Mitogen-activated protein kinases/stress-activated protein kinases
MDM2: Mouse Double Minute 2 homolog
MPS-1: Metallopanstimulin 1
NPC: Nasopharyngeal carcinoma
NRP: NPC-associated RP
PHB1: Prohibitin-1
RACK1: Receptor for Activated C Kinase 1
RASSF1A: Ras Association Domain Family 1 Isoform A
RP: Ribosomal protein
SOX4: SRY-related HMG-box
SRY: Sex-determining Region Y
TNFAIP3/A20: Tumor necrosis factor alpha-induced protein 3 or A20
USP7: Ubiquitin-specific peptidase or protease 7
